# Supplementary material for: The Experience of Prisoners with Serious Mental Disorders Participating in a Dog-Assisted Therapy Program: A Qualitative Study
Source: Animals (Basel). 2025 Jan 28;15(3):379. doi: 10.3390/ani15030379 (PMC11816068; doi:10.3390/ani15030379)
Supplement: Supplementary file 1 [file animals-15-00379-s001.zip › Supplementary S1.pdf]

Supplementary material S1

2.2. Context

The DAT program is part of the Psychosocial Support Service and Community Mediation for people with SMD, and in turn, is part of the Comprehensive Care Program for Mentally Ill Individuals in Prison *(Programa de Atención Integral al Enfermo Mental en Prisión - PAIEM)*. The PAIEM program began operating in 2009 in Spain, with the aim of providing comprehensive care to people with mental health problems in Spanish prisons, given their situation of special vulnerability (Observatory on Mental Health and Human Rights in Prisons, 2022).

The DAT program was carried out for two months with a periodicity of two days a week for 45 minutes, completing a total of 16 sessions spread over eight weeks. During the sessions, different activities focused on the intervention objectives that were developed. These included building social and communicative skills among the participants and promoting positive emotions. The dog acted as a facilitator and motivator for the fulfillment of these objectives, together with the canine guide and the expert in dog-assisted therapy.

An offer was made to all of them so that they could participate on a voluntary basis. For this, it was necessary that contact with animals was within their interests and that they were in a situation of socio-sanitary stability so as to allow a good functioning of the program, assuring the well-being of the participants and the intervention dog.

During the development of the program, two professionals with expertise in dog-assisted interventions participated as part of the human–animal team. One of them was an occupational therapist and the other was a social worker. In addition, the social worker was a canine guide with specific training in animal welfare and canine language. The occupational therapist oriented her work towards the care of people and the social worker towards the dog. The handler was responsible for detecting any stressful situation in the animal and providing the necessary help for the management of these situations, and in case the situation required it, she was the person responsible for stopping the activity and taking the dog out (a situation that never occurred).

The intervention dog (Ara) lived with her guide. In order to participate in this program, she was specially selected for her temperament and sociability and received specific training. In this type of program, the relationship of security and positive bond between the dog and its handler is of special importance. This generates a strong confidence in the dog that allows him to be authentic and to interact with people in a more joyful, secure, and positive way.

Different activities were designed where program participants had to interact with the dog. These activities were specially designed to achieve the objectives previously defined. The aim of this type of community activity is to facilitate knowledge and implementation of the access mode. It also aims to enhance communication and interpersonal skills, improve people's self-esteem and sense of self-efficacy and develop social skills by introducing individuals to new contexts of social interaction. Additionally, it can improve mood by reducing feelings of anxiety and depression and promoting positive emotions, consolidate a sense of responsibility, and foster empathy, as well as ensure adherence to the center’s activities.

Ara was a facilitator of the work so that the occupational therapist could achieve these objectives. Some examples of activities could be classified as contact activities, grooming, walking, dog sniffing, training, canine games, etc.
