# Supplementary material for: The Experience of Prisoners with Serious Mental Disorders Participating in a Dog-Assisted Therapy Program: A Qualitative Study
Source: Animals (Basel). 2025 Jan 28;15(3):379. doi: 10.3390/ani15030379 (PMC11816068; doi:10.3390/ani15030379)
Supplement: Supplementary file 1 [file animals-15-00379-s001.zip › Supplementary S2.pdf]

Supplementary material S2

2.2. Context

Session No.	Session Title	Objectives	Materials	Summary of Activities
1	Getting to Know the Dog	Foster bonding with the dog. Promote adherence to the program. Assess knowledge of the canine world and previous experiences with animals. Understand procedures and protocols of TAP programs. Encourage interaction between participants and the human and animal work team.	Treat bag, dog album, toy, water bowl, water bottle, non-slip floor.	Program presentation, rules, and protocols. Greetings and discussion about previous experiences. Individual feeding with treats. Dog's history and canine skills. Water-feedback-farewell.
2	Canine and Human Communication	Stimulate group interaction. Foster social interaction skills. Stimulate and improve verbal and non-verbal communication. Improve self-esteem.	Dog communication cards, videos, and photos.	Group greeting and dog feeding. Introduction to communication and types of communication. Activity on calming signals and dog communication. Analysis of communication in the sessions. Water-feedback-farewell.
3	Seeing the World Through Smell	Improve self-esteem. Encourage teamwork. Raise awareness about the importance of cognitive stimulation. Promote creativity and decision-making.	Treats, scent mats, scent games.	Group greeting and dog feeding. Introduction to the sense of smell and differences between dogs and humans. Testing the dog's sense of smell with materials and scent games. Water-feedback-farewell.
4	Shelters and Making Donation Materials	Gain knowledge about the world of shelters. Encourage social participation. Foster a sense of responsibility and empathy development.	Fabric strips, scissors.	Group greeting and dog feeding. Creation of dog toys with recycled materials. Donation of scent braids to a canine protection center. Water-feedback-farewell.
5	How Dogs Learn, Canine Skills	Foster bonding with the dog. Promote adherence to the program. Enhance communication and personal relationship skills. Promote teamwork. Improve self-esteem and self-efficacy.	Treat bag, dog album, toy, water bowl, water bottle, non-slip floor. Psychomotor materials (cones, hoops, poles, tunnel…).	Group greeting and dog feeding. Explanation of canine skills and group practice. Creation of a psychomotor circuit. Individual practice and rotation of positions. Water-feedback-farewell.
6	Psychomotor Circuit	Foster bonding with the dog. Promote adherence to the program. Enhance communication and personal relationship skills. Promote teamwork. Improve self-esteem and self-efficacy.	Treat bag, toy, water bowl, water bottle, non-slip floor. Psychomotor materials (cones, poles, hoops, tunnel).	Group greeting and dog feeding. Design and creation of a psychomotor circuit in a group. Execution of the circuit with the dog and group collaboration. Individual practice and circuit execution. Water-feedback-farewell.

Session No.	Session Title	Objectives	Materials	Summary of Activities
7	Cognitive Challenges, Problem Solving	Encourage teamwork. Stimulate problem-solving skills. Improve self-esteem. Exercise concentration skills.	Homemade materials found in the environment and classroom.	Group greeting and dog feeding. Introduction to canine problem-solving abilities. Creation of cognitive challenges and clues in teams for the dog. Water-feedback-farewell.
8	Partydog	Improve oral comprehension. Stimulate group interaction and communication. Develop teamwork. Encourage waiting times and active listening. Foster self-esteem. Experience pleasure from overcoming challenges.	"Partydog" board game and all previously used materials.	Group greeting and dog feeding. Interactive board game with challenges and questions, using mime and writing. Water-feedback-farewell.
9	Sharing the Experience	Improve oral comprehension. Stimulate group interaction and communication. Encourage waiting times and active listening. Share lived experiences. Experience pleasure from overcoming challenges.	Blank mural and markers.	Group greeting and dog feeding. Creation of a mural sharing experiences and emotions lived during the 2 months in contact with the dog. Water-feedback-farewell.
