# Supplementary material for: The Experience of Prisoners with Serious Mental Disorders Participating in a Dog-Assisted Therapy Program: A Qualitative Study
Source: Animals (Basel). 2025 Jan 28;15(3):379. doi: 10.3390/ani15030379 (PMC11816068; doi:10.3390/ani15030379)
Supplement: Supplementary file 1 [file animals-15-00379-s001.zip › Supplementary S3.pdf]

Supplementary material S3

The first step of the analysis occurred during data collection when researchers identified content and meanings within the data that formed part of common patterns [39]. The analysis required constant movement back and forth between the entire dataset as it was coded, analyzed, and revisited repeatedly. Thus, the analysis was not a linear process but involved jotting down ideas and developing coding schemes from the initial phase, continuing throughout the entire process, and returning to previous phases [39]. The inductive thematic analysis followed the phases described by Braun and Clarke [39]:

1. **Familiarizing with the data**: This phase requires immersion through repeated and active reading of the entire dataset, identifying meanings and common patterns. A complete reading of the data was conducted before starting the coding process, thus beginning to identify initial patterns by taking notes and jotting down ideas to revisit in later phases.
2. **Generating initial codes**: The participants' narratives were segmented, assigning a code to each narrative fragment that identified a specific characteristic of the data. Data not directly associated with a specific code but helpful for understanding the context were retained to enhance comprehension.
3. **Searching for themes**: Through consensus among researchers, the codes were organized into different categories and themes, cross-checking all excerpts of the previously identified codes using tables, as shown in the example at the end of this document, and naming and describing each theme. This process involved creating various thematic levels (codes that led to categories and categories to main themes). The data were categorized based on identifying recurring patterns that emerged from the entire dataset.
4. **Reviewing themes**: Once different themes were created in the previous phase, they were refined and verified to determine if the initial classification truly corresponded with the data obtained. Some themes were discarded as irrelevant, while others were divided, grouped, or merged depending on the coherence of the codes within them. Thus, all data within each theme were reviewed, identifying new common patterns within them that clearly reflected each theme. After this step, the entire dataset was re-read to identify whether any data discarded in previous phases could be categorized into one of the identified themes. At the end of this phase, internal coherence was achieved within each theme concerning the data they contained, as well as coherence among the different themes.
5. **Defining and naming themes**: The analysis continued to be refined, identifying and describing the "essence" of each theme. Once described, the excerpts or narratives of each category were revisited and organized coherently to accompany the description of each theme. A more detailed description of the most relevant aspects of each theme was then developed, ensuring they did not overlap while also considering each theme in relation to others and identifying the different categories. At this point, the themes were named concisely to represent a clear idea of the content of each identified theme.

6. **Producing the report**: Once all themes were identified, refined, and named, the story of the data was narrated logically and coherently, accompanied by examples from the narratives that represented the participants' lived experiences. This addressed the research question and discussed the findings in relation to the existing literature.

Examples of participants' narratives

Participant	Narrative	Code	Category	Theme
Participant 3	*"Before you even walk through the door, the animal comes out to see you, recognizes you and all of this is gratifying. It brings you joy, and you see how the animal rejoices too, as soon as she sees you. Anything here is amplified, it's something special."*	Experiencing mutual joy with the animal	Experiencing positive feelings	*Theme 1: The emotional impact of participating in the DAT program*
Participant 6	*"The mood, maybe I was feeling cross, but I got over it, I was feeling bad for whatever reason, and then I came out better. Very gratifying."*	Improvement in mood and a sense of gratification		
Participant 16	*"The dog is happy and content too, and this is contagious. The beauty of the animal, how sweet she is, the fur, how well cared for the animal is... all of that gives you happiness."*	The dog shares its happiness with you		
Participant 11	*"You spend the week better, you're happier in the module and you know that next week she comes again [the dog] (…) when I'm going to go to our session, I say to myself <<I'm going to see the animals, what fun!>>"*	Happiness and fun		
Participant 2	*"It's a bond of unconditional, selfless love, and that's not easy to find in normal life. It makes me feel affection for the animal and that affection makes me feel good towards myself (…) the dog does not judge you, and seeing how the animal behaves with you, it gives you an understanding of how a person should behave with another person, it is much more pure and healthy"*	Bond of unconditional love and affection	Bonding with the dog	
Participant 3	*"You have become an unwanted person, so a token of affection, no matter if it is from an animal, is priceless and has a psychological and emotional impact."*	Receiving affection from the dog has a positive impact		
Participant 9	*"There are many people here who only think about how to get money out of you and that makes you isolate yourself; however, you counteract that negativity with the presence of an animal that is faithful, that gives you affection."*	Counter negativity with loyalty and affection		
Participant 14	*"In prison we have shortcomings, and there is so much love, harmony, and respect that we also internalize it."*	Internalize love, harmony, and respect		

Participant 16	*"Here we are lacking in affection. Affection here is difficult, who gives us affection? Affection is given to us by our family, our loved ones, our animals. The little dog has given me much affection."*	Receiving affection from the dog that they cannot get from their loved ones		
Participant 1	*"Both regarding the animal and the therapists who provide the therapy, because it strengthens that bond. The relationship has always been very good, close, and pure trust."*	Positive relationship with therapists: closeness and trust	Feeling helped by the therapists guiding the sessions	
Participant 3	*"With them I have always had a good feeling, empathy, and reciprocity, we have understood each other very well. I have felt listened to, helped, and it's one more thing to be grateful for."*	Feeling understood, heard, and supported by the therapists		
Participant 9	*"Because I have always liked dogs. I haven't petted a puppy for eleven years, and the truth is, I've really enjoyed it."*	Fondness for dogs	The decision to participate in the program	*Theme 2: The process of participating in the DAT program*
Participant 8	*"Just to avoid being here... sitting around, you have to go to school and go to programs and do things, so you aren't thinking about anything else because if not, being in prison is more difficult, it's better to be occupied."*	Seeking distraction and engagement		
Participant 1	*"It has opened doors to things I don't know, to therapies I'm not familiar with yet, and that I'm willing to try to see how it goes, what the experience is like... driven by the desire to learn new things."*	Desire to explore new experiences and learn new things		
Participant 15	*"The first session I even got a little excited, I couldn't believe it, I said <<wow, how amazing!>>, in the end, they let them bring in the dog"*	Feeling excited because they were allowed to bring the dog	From initial surprise and disbelief to the need for incorporation as therapy	
Participant 16	*"I thought it was a joke that you were going to bring the dog; I didn't believe it. The dogs that come here are to see if people are bringing drugs. When I saw the dog, I said <<oh my God, so it's real! >> My hair stood on end."*	From disbelief to reality		

Participant 1	*"They should seriously consider implementing it and making it available in all facilities. They should introduce it because, as I said, it's a release valve, especially for the participants"*	Advocate for broader implementation		
Participant 2	*"I think it's very positive, and I believe many people [inmates from other prisons] should experience what my companions and I have felt in these sessions, because it broadens one's perspectives on life and everything"*	Extend it to inmates in other prisons		
Participant 3	*"At the beginning I was not very eager, very willing, but as the sessions went by, I was looking forward to the day, because the truth is that it gives you an extra something."*	From a lack of enthusiasm to looking forward to the session day	Progressive changes in their experience	
Participant 6	*"At the beginning I was more self-conscious and then I became more confident, both with the animal and with myself, my colleagues and the group."*	From feeling self-conscious to more confident		
